# Echocardiographic evaluation of heart in chronic obstructive pulmonary disease patient and its co-relation with the severity of disease

**DOI:** 10.4103/0970-2113.80321

**Published:** 2011

**Authors:** N. K. Gupta, Ritesh Kumar Agrawal, A. B. Srivastav, M. L. Ved

**Affiliations:** *Department of Tuberculosis and Chest Diseases, R N T Medical College, Udaipur, Rajasthan, India*

**Keywords:** Chronic obstructive pulmonary disease, cor pulmonale, echocardiography, pulmonary hypertension

## Abstract

**Background:**

Chronic obstructive pulmonary disease (COPD) has considerable effects on cardiac functions, including those of the right ventricle, left ventricle, and pulmonary blood vessels. Most of the increased mortality associated with COPD is due to cardiac involvement. Echocardiography provides a rapid, noninvasive, portable, and accurate method to evaluate the cardiac changes.

**Aims:**

To assess the cardiac changes secondary to COPD by echocardiography and to find out the correlation between echocardiographic findings and severity of COPD, if there is any.

**Materials and Methods:**

A total 40 of patients of COPD were selected and staged by pulmonary function test (PFT) and evaluated byechocardiography.

**Results:**

On echocardiographic evaluation of COPD, 50% cases had normal echocardiographic parameters. Measurable tricuspid regurgitation (TR) was observed in 27/40 cases (67.5%). Pulmonary hypertension (PH), which is defined as systolic pulmonary arterial pressure (sPAP)> 30 mmHg was observed in 17/27 (63%) cases in which prevalence of mild, moderate, and severe PH were 10/17 (58.82%), 4/17 (23.53%), and 3/17 (17.65%), respectively. The frequencies of PH in mild, moderate, severe, and very severe COPD were 16.67%, 54.55%, 60.00%, and 83.33%, respectively. Right atrial pressure was 10 mmHg in 82.5% cases and 15 mmHg in 17.5% cases. Cor pulmonale was observed in 7/17 (41.17%) cases; 7.50% cases had left ventricle (LV) systolic dysfunction and 47.5% cases had evidence of LV diastolic dysfunction defined as A ≥ E (peak mitral flow velocity of the early rapid filling wave (E), peak velocity of the late filling wave caused by atrial contraction (A) on mitral valve tracing) Left ventricle hypertrophy was found in 22.5% cases.

**Conclusion:**

Prevalence of PH has a linear relationship with severity of COPD and severe PH is almost associated with cor pulmonale. Echocardiography helps in early detection of cardiac complications in COPD cases giving time for early interventions.

## INTRODUCTION

Chronic obstructive pulmonary disease (COPD), defined by GOLD as a preventable and treatable disease with some significant extrapulmonary effects, is a very common clinical entity in clinical practice. COPD is a leading cause of death and disability worldwide. According to World Bank data it is expected to move from its status in 2000 as the 4^th^ and 12^th^ most frequent cause of mortality and morbidity, respectively, to the 3^rd^ and 5^th^ leading cause of mortality and morbidity, respectively, in 2020.[1,2] COPD is associated with significant extrapulmonary (systemic) effects among which cardiac manifestations are most common. Cardiovascular disease accounts for approximately 50% of all hospitalization and nearly one third of all deaths, if forced expiratory volume in one second (FEV_1_)> 50% of predicted.[3] In more advanced disease cardiovascular disease account for 20%–25% of all deaths in COPD.[4] COPD affects pulmonary blood vessels, right ventricle, as well as left ventricle leading to development of pulmonary hypertension, cor pulmonale, right ventricular dysfunction, and left ventricular dysfunction too. Echocardiography provides a rapid, noninvasive portable and accurate method to evaluate the right ventricle function, right ventricular filling pressure, tricuspid regurgitation, left ventricular function and valvular function.[5] Many studies have confirmed that echocardiographically derived estimates of pulmonary arterial pressure co-relate closely with pressures measured by right heart catheter (*r*> 0.7).[6,7]

Hence the present study was undertaken with the following aims and objectives:

1. To assess the cardiac changes secondary to COPD by echocardiography, and
2. To find out the correlation between echocardiographic findings and the severity of COPD using GOLD guidelines.

## MATERIALS AND METHODS

Forty patients of COPD confirmed by clinical history, radiology of chest, and pulmonary function test were selected from Chest and T.B. Hospital of R.N.T. Medical College, Udaipur, Rajasthan. During selection, patients with H/O of chronic lung disease other than COPD, hypertension, any primary cardiac disease, any systemic disease that can cause pulmonary hypertension, patients with poor echo window, and patients who were unable to perform spirometry were excluded from the study.

All selected patients were subjected to routine investigations, including complete blood count, lipid profile, blood sugar, blood urea, serum creatinine, electrocardiography, and so on, as needed.

All the patients were investigated by spirometry and diagnosed and classified according to GOLD guidelines (postbronchodilator FEV _1_ /forced vital capacity (FVC) ratio < 70% predicted), mild (FEV _1_ ≥ 80% of predicted), moderate (50% ≤ FEV _1_ < 80% predicted), severe (30% ≤ FEV _1_ < 50% predicted), and very severe (FEV _1_ < 30% predicted), respectively.

All patients were subjected to resting two-dimension transthoracic Doppler echocardiography in the cardiology department of R.N.T. Medical College and associated hospitals by expert cardiologists. The machine used was VIVID 7 model of GE health care system with a multifrequency probe with a range of 2–4.3 MHz. Both 2D and M-Mode studies were done.

Echocardiography was reviewed to assess the pericardium, valvular anatomy and function, left and right side chamber size and cardiac function. Tricuspid regurgitant flow was identified by color flow Doppler technique and the maximumjet velocity was measured by continuous wave Doppler withoutthe use of intravenous contrast. Right ventricular systolicpressure was estimated based on the modified Bernoulli equationand was considered to be equal to the sPAP in the absence ofright ventricular outflow obstruction: sPAP (mmHg) = rightventricular systolic pressure = trans-tricuspid pressure gradient (TTPG) + rightatrial pressure (RAP), where trans-tricuspid gradient is 4*v*^2^ (*v* = peak velocity of tricuspid regurgitation, m/s).[6,8,9] RAP was empirically estimated as 15 mmHg before 1997. Since1997, RAP was estimated to be 5, 10, or 15 mmHg based on thevariation in the size of inferior vena cava with inspirationas follows: complete collapse, RAP = 5 mmHg; partial collapse, RAP = 10 mmHg; and no collapse, RAP = 15 mmHg.[10]

Pulmonary hypertension (PH) was defined in this study as sPAP ≥ 30 mmHg.[11] This value was chosen according to the definition of pulmonary hypertension. PH was classified into mild, moderate, and severe category as sPAP 30–50, 50–70,>70 mmHg, respectively (using Chemla formula, mean pulmonary arterial pressure (MPAP) =0.61 PASP + 2 mmHg and putting value of 25–35, 35–45, and>45 mmHg of MPAP for mild, moderate, and severe pulmonary hypertension, respectively).[12]

Right ventricle dimension was measured by M-Mode echo and right ventricular dilation or cor pulmonale was said to be present when it exceeded the normal range of 0.9–2.6 cm. Right ventricle contractility was also noted and right ventricular systolic dysfunction was said to be present when it was hypokinetic.

Left ventricular function was also assessed by using the following parameters: EF (ejection fraction) = measure of how much end-diastolic value is ejected from LV with each contraction (56%–78%)

FS (fractional shortening) = it is a percentage change in LV dimension with each LV contraction (28%–44%).

LV mass = left ventricular mass (88–224 g).

E/A = diastolic filling of left ventricles usually classified initially on the basis of the peak mitral flow velocity of the early rapid filling wave (E), peak velocity of the late filling wave caused by atrial contraction (A). In normal subjects LV elastic recoil is vigorous because of normal myocardial relaxation, therefore more filling is completed during early diastolic, so left ventricular diastolic dysfunction (LVDD) is said to be present when E/A is <1.3 (age group 45–49 years), <1.2 (age group 50–59 years), <1.0 (age group 60-69 years), and <0.8 (age group ≥70 years).[13]

## RESULTS

A total of 40 patients were recruited in our study and out of them, the number of patients with mild, moderate, severe, and very severe COPD were 18/40 = 45%, 11/40 = 27.5%, 5/40 = 12.5%, and 6/40 = 15%, respectively [Table 1]

**Table 1:** Patients classification according to severity of COPD

Severity of COPD	No. of patients	% Patients
Mild (FEV1 > 80% predicted)	18	45
Moderate (50% > FEV1<80% predicted)	11	27.50
Severe (30%< FEV1 < 50% predicted)	5	12.50
Very severe (FEV1 < 30% predicted)	6	15

Majority of cases belong to mild COPD. FEV_1_: Forced expiratory volume in one second; COPD: Chronic obstructive pulmonary disease

On echocardiography 20 patients (20/40 = 50%) had normal study.

Measurable tricuspid regurgitation (TR) was observed in 27 patients (27/40 = 67.5%). Mean TTPG for the entire group in which TR could be measured was 37.04 mmHg.

PH defined as sPAP> 30 mmHg was observed in 17 patients (17/40 = 42.5% of the total study population) (17/27 = 63% of measurable TR). Out of those 17 patients with pulmonary hypertension, 10 patients were in mild PH (sPAP 30-50 mmHg) (10/40 = 25%) (10/17 = 58.82%), 4 were in moderate PH (sPAP 50-70 mmHg) (4/40 = 10%) (4/17 = 23.53%), and 3 were in severe PH (sPAP> 70 mmHg) (3/40 = 7.5%) (3/17 = 17.65%) [Table 2]. The frequencies of PH in mild, moderate, severe, and very severe COPD were 3/18 = 16.67%, 6/11 = 54.55%, 3/5 = 60%, 5/6 = 83.33%, respectively; thus we can see that there is a good co-relation between the frequency of PH and severity of COPD [Table 3].

**Table 2:** Echocardiographic findings in our study

Findings	No. of patients	% Patients
Normal study	20	50
Pulmonary hypertension		
(sPAP > 30 mmHg)	17	42.50
Mild (30-50 mmHg)	10	25
Moderate (50--70 mmHg)	4	10
Severe (>70 mmHg)	3	7.50
Cor pulmonale	7	17.50
RVSD	3	7.50
LVH	9	22.50
LVDD	19	47.50
LVSD	3	7.50

Besides PH and cor pulmonale, left ventricular dysfunction is also common. RVSD: Right ventricular systolic dysfunction; LVH: Left ventricular hypertrophy; LVDD: Left ventricular diastolic dysfunction; LVSD: Left ventricular systolic dysfunction

**Table 3:** Frequency of PH with severity of COPD

Severity of COPD	% and number of patients with PH
Mild (18)	16.7% (3)
Moderate (11)	54.6% (6)
Severe (5)	60% (3)
Very severe (6)	83.3% (5)

Frequency of PH increases as the severity of COPD

The frequencies of cor pulmonale in patients with mild, moderate, and severe PH were 10%, 75%, and 100%, respectively; so we can see a good co-relation between severity of PH and the development of cor pulmonale [Tables 4 and 5].

**Table 4:** Frequency of cor pulmonale with severity of COPD

Severity of COPD	% and number of patients with PH
Mild (18)	11.11% (2)
Moderate (11)	9% (1)
Severe (5)	40% (2)
Very severe (6)	33.33% (2)

NO co-relation exists between frequency of cor pulmonale and severity of COPD

**Table 5:** Frequency of cor pulmonale with severity of PH

Severity of PH	Frequency of cor pulmonale
Mild (10)	10% (1)
Moderate (4)	75% (3)
Severe (3)	100% (3)

Frequency of cor pulmonale increases with severity of PH

Comparative study of various stages of severity of COPD reveals that as severity of COPD increases the prevalence of cardiac dysfunction increases, so more severe COPD is associated with more prevalent and more severe cardiac manifestations [Table 6].

**Table 6:** Echocardiographic findings according to severity of COPD

	Mild (18)	Moderate (11)	Severe (5)	Very severe (6)
Normal	13	5	2	0
PH	3	6	3	5
Cor pulmonale	2	0	3	2
RHF	1	0	1	1
LVDD	6	4	3	6
LVH	3	2	2	2
LVSD	1	0	1	1

As severity of COPD increases the prevalence of cardiac dysfunction also increases. COPD: Chronic obstructive pulmonary disease; PH: Pulmonary hypertension; RHF: Right heart failure; LVDD: Left ventricular diastolic dysfunction; LVH: Left ventricular hypertrophy; LVSD: Left ventricular systolic dysfunction

## DISCUSSION

The cardiac manifestations of COPD are numerous. Impairment of right ventricular dysfunction and pulmonary blood vessels are well known to complicate the clinical course of COPD and co-relate inversely with survival. Significant structural changes occur in the pulmonary circulation in patients with COPD. The presence of hypoxemia and chronic ventilator insufficiency is associated with early evidence of intimal thickening and medial hypertrophy in the smaller branches of the pulmonary arteries. Coupled with these pathological changes are pulmonary vasoconstriction arising from the presence of alveolar hypoxemia, destruction of pulmonary vascular bed, changes in intrinsic pulmonary vasodilator substances (such as decrease in PGI _2_ s (prostacyclin synthase), decrease in eNOS (endothelial nitric oxide synthase), and increase in ET1 (endothelin1) leads to remodeling, increase in blood viscosity, and alteration in respiratory mechanics. All these lead to a significant increase in pulmonary vascular resistance, the consequence of which is pulmonary hypertension. Severe PH increases right ventricular after load with a corresponding increase in right ventricular work, which results in uniform hypertrophy of the right ventricle. In patients with COPD, hypoxic vasoconstriction is associated with not only right ventricular hypertrophy but also right ventricular dilation which eventually leads to clinical syndrome of right heart failure with systemic congestion and inability to adapt right ventricular output to the peripheral demand on exercise.

Although the true prevalence of PH in COPD is unknown, an elevation of pulmonary arterial pressure is reported to occur in 20%–90% of patients when measured by right heart catheterization with some evidence that pulmonary hemodynamic worsens with worsening airflow obstruction.[14–19] Two studies have shown an abnormal increase in mean pulmonary arterial pressure (Ppa) in COPD of 0.4–0.6 mmHg per year. These studies illustrate that PH in COPD progresses slowly and occurs in mild as well as severe forms of disease.[20,21]

The level of PH has a prognostic value in COPD patients that has been demonstrated by several studies. In one of these studies, the 5-year survival rates were 50% in patients with mild PH (20–30 mmHg), 30% in those with moderate-to-severe PH (30–50 mmHg), and 0% in the small group of patients with very severe PH (>50 mmHg). Thus a high degree of PH bears a poor prognosis, and this also has been observed in COPD patients receiving long-term oxygen therapy.[22] The present study finding reveals 42.5% patients of various severity of COPD have findings of pulmonary hypertension, that is similar in prevalence of previous studies. The frequencies of PH in mild, moderate, severe, and very severe COPD were 16.67%, 54.55%, 60.00%, and 83.33%, respectively. In one study it was found to be 25%, 43%, and 68% in mild, moderate, and severe COPD, respectively.[23] In our study it is also observed that severe PH is present only in severe or very severe COPD. In conclusion, the incidence of PH is directly proportional to severity of disease. Previous studies showed the frequencies of severe PH in COPD from about 1%–3%,[24,25] but in our study it is 4.7%; this may be due to small study population comprising more percentage of severe and very severe COPD patients.

Cor pulmonale is present in 17.5% of patients in our study. Approximately 25% patients with COPD eventually develop cor pulmonale.[26] Cor pulmonale was found in 40% patients with COPD in one autopsy study.[27,28] It is estimated that every year between 10% and 30% of all hospital admissions for heart failure in the United States are due to cor pulmonale[29] and approximately 85% patients with cor pulmonale have COPD.[26]

Some studies indicate that LV function remains normal in persons with COPD, whereas others suggest that LV dysfunction may be present.[30,31] Abnormal LV performance in persons with COPD may be due to a number of factors, such as hypoxemia and acidosis; concurrent coronary artery disease; ventricular interdependence (because the right ventricle (RV) and LV share a common septum, RV dilatation may lead to bulging of the septum into the LV, which would in turn increase LV end-diastolic pressure, decrease venous return, and diminish LV stroke volume and cardiac output (CO) and large swings in intrathoracic pressure (pronounced negative pleural pressure would increase Ppa and diminish LV stroke volume due to ventricular interdependence, negative pleural pressures may also increase LV after load).[32]

In our study, left ventricular systolic dysfunction (LVSD) is present in 7.5% patients, in previous studies it was present in 4%–32% patients of COPD.[33–36] LVDD was seen in COPD patients with normal pulmonary arterial pressure and it increased with right ventricular after load.[37] In our study LVDD is present in 47.5% of patients, out of which 16 patients had PH and 3 did not have PH, various mechanisms might explain the presence of left diastolic dysfunction in COPD patients. This may be due to chronic hypoxemia leading to abnormalities of myocardial relaxation, lung hyperinflation, and distension leading to increased stiffness of the parietal pleura and thus of the wall of cardiac fossa leading to added load on ventricle, and also due to ventricular interdependence.

Left ventricular hypertrophy (LVH) was present in 22.5% patients in our study, in one previous study LVH was found in 25%–60% patients dying of COPD mainly in patient who also had right ventricular hypertrophy.[38]

## CONCLUSION

To conclude, the present study shows high prevalence of pulmonary hypertension, cor pulmonale, left ventricular dysfunction complicating COPD, more so with more severe COPD. We suggest screening of all COPD patients for cardiac complications. This would contribute to the assessment of prognosis in these patients and assist in identifying individuals likely to suffer increased mortality and morbidity warranting close monitoring and intense treatment.
